# Is the Fistula First Approach still valid?

**DOI:** 10.1590/2175-8239-JBN-2020-U001

**Published:** 2021-02-26

**Authors:** Ricardo Portiolli Franco

**Affiliations:** 1 Fundação Pró-Renal Brasil, Centro de Nefrologia Intervencionista, Curitiba, PR, Brasil.

**Keywords:** Dialysis, Renal Dialysis, Fistula, Arteriovenous fistula, Nephrology, Vascular Access Devices, Vascular Grafting, Diálise, Diálise Renal, Fístula, Fístula Arteriovenosa, Nefrologia, Dispositivos de Acesso Vascular, Enxerto Vascular

## Abstract

The *Fistula First Breakthrough Initiative*, founded in 2003, was responsible for changing the access profile in the United States, increasing the prevalence of arteriovenous fistulas (AVFs) by 50% and reducing that of arteriovenous grafts (AVGs). However, the concept that AVFs are always the best access for all patients has been challenged. Discussion points are: (1) the questionable survival benefit of AVFs over AVGs, if one takes into account the high rates of primary AVF failure; (2) the potential benefits of using AVGs for greater primary success; and (3) the questionable benefit of AVFs over AVGs in patients with shorter survival, such as the elderly. The high rate of primary failure and maturation procedures leads to prolonged use of catheters, and it is one of the weaknesses of the fistula first strategy. AVGs proved to be better than AVFs as a second access after the failure of a first AVF, and in patients with non-ideal vessels, with greater primary success and reduced catheter times. AVGs appear to have a similar survival to AVFs in patients older than 80 years, with less primary failures and interventions to promote maturation. The most recent KDOQUI guidelines suggest an individualized approach in access planning, taking into account life expectancy, comorbidities and individual vascular characteristics, with the aim of chosing adequate access for the right patient, at the right time, for the right reasons.

## RESUMO

A *Fistula First Breakthrough Initiative*, fundada em 2003, foi responsável pela mudança do perfil dos acessos nos Estados Unidos, aumentando em 50% a prevalência das fístulas arteriovenosas (FAVs) e reduzindo a de enxertos arteriovenosos (EAVs). No entanto, o conceito de que as FAVs são sempre o melhor acesso para todos os pacientes vem sofrendo questionamentos. São pontos de discussão: (1) o questionável benefício de sobrevida das FAVs sobre os EAVs, se levadas em conta as altas taxas de falência primária das FAVs; (2) os potenciais benefícios no uso de EAVs pelo maior sucesso primário; e (3) o benefício questionável das FAVs sobre os EAVs em pacientes com menor sobrevida, como os idosos. A alta taxa de falência primária e de procedimentos para maturação leva ao uso prolongado de cateteres e é um dos pontos fracos da estratégia "*Fistula First*". Os EAVs mostraram superioridade em relação às FAVs como segundo acesso após a falência de uma primeira FAV e em pacientes com vasos não ideais, com maior sucesso primário e redução dos tempos de cateter. Os EAVs parecem ter sobrevida semelhante à das FAVs nos idosos acima de 80 anos, com menos falências primárias e intervenções para maturação. As diretrizes mais recentes do KDOQUI sugerem uma abordagem individualizada no planejamento dos acessos, levando-se em conta expectativa de vida, comorbidades e características vasculares individuais, com o objetivo de indicar acesso adequado para o paciente adequado, no tempo adequado, pelos motivos adequados.

## INTRODUCTION

Countless papers on vascular access for hemodialysis started with some variation of the phrase “The arteriovenous fistula (AVF) is the gold standard access for hemodialysis, due to due to it’s lower rate of complications and mortality compared to arteriovenous grafts (AVGs) and catheters”. The major responsible for raising awareness on the use of AVF as the access of choice was the Fistula First Breakthrough Initiative (FFBI). The FFBI played an essential role in changing the profile of vascular accesses in many services, creating the culture necessary for the change from mostly AVG use for a majority of fistulas, mainly in the United States of America (USA).

The increase in the creation of AVFs has shown a rate of primary of maturation failures from 23% to 46%, with a great need for interventions to reach maturity.^1^^-^ 5 The use of catheters as a bridge for a new AVF has become frequent, and AVGs were reserved for patients without the possibility of having an AVF. Knowledge in vascular access has advanced, and the evidence challenges the dogma that an AVF is always the best access for all patients.

In this paper, we review the evolution of the concepts proposed by the FFBI and its current application in the planning of vascular accesses.

## DISCUSSION

### FFBI CONCEPTS AND IMPACT

In the 1990s, hemodialysis services in the USA had a huge number of patients with AVGs, an accesses that requires a greater number of interventions to maintain patency and with a higher incidence of infections.^6^ The FFBI addressed this problem through eleven concepts, with the recent addition of the last two (Table 1). The FFBI’s focus was limited to: (1) continuous data collection and reassessment; (2) early referral for the creation of an AVF in a timely manner; (3) creation of an AVF as the access of choice. The initial objective, in 2003, was to reach 40% of AVFs in incident patients and 50% in prevalent patients. This goal was achieved in 2005, with a 50% increase in the prevalence of AVFs in the USA, and a new target was set, 66% of AVFs by 2009.^7^^,^ 8

**Table 1:** Fistula First Breakthrough Initiative Concepts

1	Routine Continuous Quality Improvement (CQI) review of vascular access
2	Timely referral to nephrologist
3	Early referral to surgeon for “AVF only” evaluation and timely placement
4	Surgeon selection based on best outcomes, willingness, and ability to provide access services
5	Full range of surgical approaches to AVF evaluation and placement
6	Secondary AVF placement in patients with AVGs
7	AVF placement in patients with catheters where indicated
8	AVF cannulation training
9	Monitoring and maintenance to ensure adequate access function
10	Education for caregivers and patients
11	Outcomes feedback to guide practice
12	Modify hospital systems to detect CKD and promote AVF planning and placement
13	Support patient efforts to live the best possible quality of life through self-managemen

Source: Lee T. Fistula First Initiative: historical impact on vascular access practice patterns and influence on future vascular access care (Adapted).^7^

AVF: arteriovenuous fístual; AVG: arterial-venous graft; CKD: chronic kidney disease.

After the FFBI, there was a significant increase in the prevalence of AVFs. In 2003, the USRDS showed an AVF prevalence of 32%, AVG of 40% and 27% catheters. In 2018, the USRDS showed 63% of patients with AVFs, 17.5% with AVGs and 19.6% with catheters.^9^The increase in AVFs occurred mainly due to the reduction in AVGs, with a proportionally lower impact on the reduction of catheters. For this reason and because of the concern with catheter-related morbidity, the initiative adopted the slogan “Fistula first, catheter last” in 2014.

### “FISTULA FIRST”, ALWAYS?

Important points of discussion about the indication of AVFs for all patients are: (1) the questionable advantage regarding the survival of AVFs over AVGs, apparent in publications due to the exclusion of frequent primary failures in survival analyzes; (2) the possible benefit of AVGs as a bridge access due to greater primary success; and (3) the questionable benefit of AVFs over AVGs in patients with shorter survival, such as the elderly.

KDOQI 2006 already incorporated FFBI concepts into its guidelines; however, the group emphasized that “in some cases, the “Fistula First Approach at all costs” may not be cost-effective or optimal for each individual”. The guidelines reinforced that the objective should be a functional AVF, not just the creation of an AVF, and cited AVGs as a bridge to a secondary AVF, to reduce catheter time.^10^

In 2007, Lok reported the potential increase in catheter use due to the use of AVF as the access of choice for all patients, and questioned the survival advantage of AVFs over AVGs, mainly due to the exclusion of primary failures in survival comparisons.^11^^,^ 12 When primary failures are excluded from survival analyzes, mature AVFs and AVGs are compared, and in this comparison the patency of AVFs is actually greater.^3^^,^ 13 However, there appears to be no difference in the secondary patency between AVFs and AVGs when we include primary failures in the analyzes.^3^^,^ 14

The relatively low probability of achieving a functional AVF in a short period, without additional interventions and the permanence of catheters with repeated attempts, is a weakness of the fistula first strategy. A 10-year USRDS review, with 1740 acesses, showed twice as many primary failures in AVFs as compared to AVGs (39.7% *vs*. 18.8%, *p* < 0.001), as first or second access.^3^ A meta- analysis reported 3.4 months as average time for AVF maturation, with 66% of patients requiring catheter use, and approximately 20% abandoning the access without using it for hemodialysis.^15^ While only 20% of patients in general remain with a catheter after an AVF maturation; in cases of primary failure, up to 65% still use a catheter after eight months, and only 19% use an AVG or AVF.^16^ Comparably, evaluating patients who were submitted exclusively to AVF, only 57% of these lead to catheter independence and, even with multiple attempts, only 40% of the time on hemodialysis is catheter-free.^17^ However, these AVF weaknesses, do not totally overcome their long-term benefits, because a mature and functional AVF has less need for interventions and infections compared to AVGs, and probably benefits the majority of patients. Therefore, the criticism of the indication of AVF as the best access always lies in its placement in patients with characteristics that are not favorable to the maturation of AVFs, such as small arteries and veins, and in the increase in exposure to catheters due to the high chance of primary and maturation failures.

### THE ROLE OF AVGS

A randomized controlled study comparing radiocephalic AVFs and forearm AVGs in patients with non-ideal vessels (radial artery between 1 and 2 mm and cephalic vein < = 1.6 mm) showed AVG superiority (primary patency 33% *vs*. 44%, *p* = 0.03; and secondary 52% *vs*. 79%, *p* = 0.001), despite the greater need for thrombectomies. The rate of primary AVF failure was 41% and that of AVG, only 2%.^18^ In patients with a first radiocephalic AVF failure, AVGs led to shorter catheter times and less cathter related infections compared to brachiocephalic AVFs in a retrospective analysis.^19^ AVGs had a higher primary and secondary patency in relation to brachiocephalic AVFs, when including primary AVF failures, which were twice as common (10 *vs*. 26, *p* = 0.006).

Since AVGs have a higher rate of primary success and can be cannulated earlier, it is interesting to consider them as initial access in catheter-dependent patients.^20^ This strategy can be useful in the case of patients with poor vascular conditions, with low probability of AVF maturation.

### KDOQI 2019

These questions led to changes in the KDOQI in 2019.^21^ The guidelines suggest an individualized “Life Plan in CKD”, updated annually and documented in medical records that contemplates not only the first access, but a contingency strategy in case of dysfunction and planning of the next accesses, if there is irreversible failure. The guidelines recommend that the incident or prevalent patient on hemodialysis preferably have an AVF or AVG instead of a catheter, due to the lower risk of infection and hospitalizations associated with these accesses, when consistent with their treatment goals. The text reinforces the lack of strong evidence to choose a particular type of access based solely on reduced mortality. The choice between AVG and AVF as initial or substitute access to catheters depends on clinical judgment, vascular characteristics, chance of maturation of the different types of AVF, comorbidities, life expectancy and patient choice. Table 2 compares selected guidelines from the last two editions of KDOQI. In patients with an estimated survival of less than one year, the latest guidelines consider AVG or AVF with a high chance of maturation (i.e. brachiocephalic) as the first choice. In patients with an estimated survival of more than one year, AVG are an option for those with a low probability of primary AVF maturation, in order to anticipate the removal of the catheters. After this first “bridge” AVG fails, the placement of a secondary AVF is considered. The comparison of the two KDOQI editions highlights the current emphasis on individualizing access planning and the conscious indication of the “adequate access for the right patient, at the right time, for the right reasons” moving away from “better access for all” approach.^21^ The KDOQI group evaluated evidence published until October 2016.

**Table 2:** Comparison of guidelines selected from KDOQI 2006 and 2019

KDOQI 2006		
Time of Fistula Making	Access place and type principles	
AVF 6 months after the HD onset AVG 3 to 6 weeks before the HD	Distal antes de proximal FAV antes de EAV Non-dominant side before the dominant one	Order of preference: 1. Wrist radiocephalic AVF 2. Brachiocephalic AVF 3. Transposition brachiocephalic AVF 4. Forearm loop AVG 5. Upper limb AVG 6. Lower limb collar AVF/AVG
**KDOQI 2019**		
**Time of fistula making**		**Access place and type principles**
GFR 15 - 20 mL/min/1.73m2 Life on CKD plan and individualized treatment goal. Consider AVF if maturation time and conditions are adequate.		Access:
		• HD estimate duration (more or less one year).
		• Maturation probability of each type of access.
		• Catheter use and HD urgent onset.
**CKD life plan and Access planning for incident patients**		
	HD expectancy > 1 year	HD expectancy < 1 year
Non-urgent onset	Algorithm 1 1. Distal AVF 2. Proximal AVF OR forearm AVG 3. Brachiobasilic or proximal AVF	1. Forearm AVG or brachiocephalic AVF (with a high likelihood of unassisted maturation). 2. Proximal AVG
Urgent onset	1. PD. If not a long term option - follow algorhitm (1) 2. Forearm early puncture AVG. After failure, follow algorithm (1). 3. Catheter if high likelihood of rapid AVF maturation and usability success, then follow algorithm (1).	1. AVG or catheter*

Source: Lok CE, Huber TS, Lee T, et al. KDOQI Clinical Practice Guideline for Vascular Access: 2019 Update. Am J Kidney Dis. 2020;75(4):S1-S164 (Adaptado).^21^

HD: hemodiálise; TFG: taxa de filtração glomerular; FAV: Fístula arteriovenosa; EAV: Enxerto arteriovenoso; DP: diálise peritoneal.

### EARLY CANNULATION AVG

Studies with early cannulation AVGs, which can be cannulated within 24 hours after implantation, point to potential advantages. A randomized study with 121 patients starting urgent hemodialysis showed less bacteremia and hospitalizations in the early cannulation AVGs group compared to tunneled catheters, with no difference in total costs.^22^ However, only 23% in the AVG group and 16% in the catheter group were dialyzed an AVF after 6 months, leading to questions in the planning of accesses in the follow-up.^23^ KDOQI 2019 considers the use of these AVGs as a possible strategy in reducing catheter use.^21^ A retrospective analysis published after KDOQI compared AVF (n = 131) and early cannulation AVG (n = 266) in patients starting urgent hemodialysis. No patient in the AVG group needed a catheter, compared to all of them in the AVF group. The AVG had better assisted primary patency (47.8% *vs*. 76.2%, *p* < 0.001) and secondary patency in two years (63.3% *vs*. 81.2% *p* < 0.001), less exposure to catheters (14235 *vs*. 3650 catheters/day, *p* < 0.01) and sepsis (42 *vs*. 4, *p* < 0.01). The AVG group still had lower mortality after one year of follow-up (15.2% *vs*. 21.6%, *p* = 0.034). Although there were more interventions to maintain patency in AVGs, the costs were significantly lower, mainly due to the lower rate of complications related to catheters.^24^ The use of early cannulation AVGs can be an alternative to catheters; however, controlled randomized trials are needed, as well as an assessment of which group of patients will benefit from this strategy.

### ACCESS IN THE ELDERLY

The increase in the elderly population on dialysis represents a challenge for access planning. Because these patients often have higher mortality, worse vascular conditions and more comorbidities, the insistent pursuit for an AVF as the access of choice can generate multiple interventions without success.

Patients over 77 years of age had a higher chance of primary failure (HR 1.19, 95% CI 1.25 -1.45) and need for assisted maturation in 55% of cases (OR 1.12 95% CI 1.05 - 1, 21), compared to those aged 67 to 77 in a retrospective assessment of more than 22,000 patients.^25^ Accesses that require interventions to mature in the elderly also have greater need of new interventions.^26^ However, other recent retrospective studies suggests AVF patency is not affected by age.^16^^,^ 17 ^,^ 25

A retrospective assessment in the elderly showed similar survival among patients over 80 years of age who had an AVG or AVF as their first pre-dialysis access, with worse survival in those starting with a catheter.^27^ These findings were repeated in a recent analysis, with similar patency outcomes between AVF and AVG in the group over 80 years old, but with an advantage for AVFs, including patient survival, in the groups from 65 to 79 years of age.^28^

Age should not be used alone as an exclusion factor for AVF; however, due to the shorter survival of elderly patients on dialysis, the patient’s option should be specially considered and the indication for an AVF placement individualised, for those with good vascular conditions and good chances of maturation. In the case of patients over 80 years old, AVGs seem to be an interesting choice, which can reduce the time of catheter use and provide survival equivalent to that of AVF.

## CONCLUSIONS

The FFBI played an essential role in raising awareness and implementing the AVF as the access of choice. However, the “best access for all” approach seems questionable, and the clinical judgment, taking into account comorbidities, life expectancy and individual vascular characteristics, helps in deciding on the best access for each patient. AVGs can reduce exposure to catheters and the number of interventions in patients with a low chance of AVF maturation or with low life expectancy. Does the fistula firsrt approach still valid? Not always nor for all patients, but yes when conditions for maturation are favorable and the long term benefits are considerable.
