# Erratum

**DOI:** 10.1590/2175-8239-JBN-2020-U001er

**Published:** 2021-11-01

In the article “Fistula First Approach: still valid?”, with DOI code number https://doi.org/10.1590/2175-8239-JBN-2020-U001, published at Brazilian Journal of Nephrology, 2021;43(2):263-268:

Where it was written:

**Page 264:**

Countless papers on vascular access for hemodialysis started with some variation of the phrase “The arteriovenous fistula (AVF) is the gold standard access for hemodialysis, **due to due to** it’s lower rate of complications and mortality compared to arteriovenous grafts (AVGs) and catheters”.

The increase in the creation of AVFs has shown a rate **of primary of maturation** failures from 23% to 46%, with a great need for interventions to reach maturity.1–5

**Page 265 (Table 2):**

Distal antes de proximal.

FAV antes de EAV.

HD: hemodiálise, TFG: taxa de filtração glomerular, FAV Fístula arteriovenosa, EAV Enxerto arteriovenoso, DP: diálise peritoneal.

Should read:

**Page 264**:

Countless papers on vascular access for hemodialysis started with some variation of the phrase “The arteriovenous fistula (AVF) is the gold standard access for hemodialysis, **due to** it’s lower rate of complications and mortality compared to arteriovenous grafts (AVGs) and catheters”.

The increase in the creation of AVFs has shown a rate **of primary or maturation** failures from 23% to 46%, with a great need for interventions to reach maturity.1–5

**Page 265 (Table 2)**:

Distal before proximal.

AVF before AVG.

HD: hemodialysis, GFR: glomerular filtration rate, AVF arteriovenous fistula, AVG arteriovenous graft, PD: peritoneal dialysis.

