# Errata: Abordagem Fistula First: ainda válida?

**DOI:** 10.1590/2175-8239-JBN-2020-U001er2

**Published:** 2022-05-25

No artigo “**Abordagem Fistula First: ainda válida?**”, com o DOI: https://doi.org/10.1590/2175-8239-JBN-2020-U001, publicado no Brazilian Journal of Nephrology, 2021;43(2):263-268 na versão em português:

Onde está escrito:

**Tabela 2:** Comparação de diretrizes selecionadas dos KDOQI 2006 e 2019

Plano de vida na DRC e Planejamento de acessos em pacientes incidentes		
	Expectativa de HD < 1 ano	Expectativa de HD > 1 ano
Início não urgente	Algoritmo 1 1. FAV distal 2. FAV proximal OU EAV de antebraço 3. FAV braquiobasílica ou EAV proximal	1. EAV de antebraço ou FAV braquiocefálica (com alta chance de maturação não assistida). 2. EAV proximal
Início urgente	1. DP. Se não for opção em longo prazo, seguir algoritmo (1) 2. EAV de punção precoce em antebraço. Após falência, seguir algoritmo (1). 3. Cateter se a probabilidade de maturação rápida e uso de uma FAV forem altas, então seguir algoritmo 1.	1. EAV ou cateter*

Leia-se:

**Tabela 2:** Comparação de diretrizes selecionadas dos KDOQI 2006 e 2019

Plano de vida na DRC e Planejamento de acessos em pacientes incidentes		
	Expectativa de HD **>** 1 ano	Expectativa de HD **<** 1 ano
Início não urgente	Algoritmo 1 1. FAV distal 2. FAV proximal OU EAV de antebraço 3. FAV braquiobasílica ou EAV proximal	1. EAV de antebraço ou FAV braquiocefálica (com alta chance de maturação não assistida). 2. EAV proximal
Início urgente	1. DP. Se não for opção em longo prazo, seguir algoritmo (1) 2. EAV de punção precoce em antebraço. Após falência, seguir algoritmo (1). 3. Cateter se a probabilidade de maturação rápida e uso de uma FAV forem altas, então seguir algoritmo (1).	1. EAV ou cateter*

